# Borealpox [bōr′-ē-әl-poks]

**DOI:** 10.3201/eid3205.241377

**Published:** 2026-05

**Authors:** JJ L. Miranda

**Affiliations:** Barnard College, Columbia University, New York, New York, USA

**Keywords:** Borealpox, viruses, poxviridae, orthopoxvirus, zoonoses, Alaska, taiga, etymology, United States

The emerging borealpox virus causes zoonotic borealpox disease, characterized by dermal lesions, in humans. From the Latin adjective *borealis*, which refers to Boreas (βορέας), the Greek god of the north wind, boreal has indicated northern origin since the 15th Century. 

The orthopoxvirus isolate AK2015_poxvirus was isolated from a resident of Fairbanks, Alaska, USA, in 2015, and initially dubbed Alaskapox virus after complete genome sequence analysis revealed a novel species similar to Old World orthopoxviruses. Seven human cases, including 1 fatal case, preceded renaming to borealpox virus. Boreal references the taiga, a subarctic coniferous forest ecosystem where case-patients and likely small mammal virus reservoirs resided.
